# Informing interventions to improve uptake of adjuvant endocrine therapy in women with breast cancer: a theoretical-based examination of modifiable influences on non-adherence

**DOI:** 10.1007/s00520-023-07658-x

**Published:** 2023-03-04

**Authors:** Caitriona Cahir, Kathleen Bennett, Stephan U. Dombrowski, Catherine M. Kelly, Mary Wells, Eila Watson, Linda Sharp

**Affiliations:** 1 Data Science Centre, School of Population Heath Sciences, RCSI University of Medicine and Health Sciences, Dublin, Ireland; 2 Faculty of Kinesiology, University of New Brunswick, Fredericton, Canada; 3 Mater Private Hospital, Dublin, Ireland; 4 Imperial College Healthcare NHS Trust, Imperial College London, London, UK; 5 Oxford Institute of Nursing, Midwifery and Allied Health Research (OxINMAHR), Faculty of Health and Life Sciences, Oxford Brookes University, Oxford, UK; 6 Population Health Sciences Institute, Newcastle University Centre for Cancer, Newcastle University, Newcastle upon Tyne, UK

**Keywords:** Adherence, Breast cancer, Endocrine therapy, Intervention development

## Abstract

**Purpose:**

To inform intervention development, we measured the modifiable determinants of endocrine therapy (ET) non-adherence in women with breast cancer, using the Theoretical Domains Framework (TDF) and examined inter-relationships between these determinants and non-adherence using the Perceptions and Practicalities Approach (PAPA).

**Methods:**

Women with stages I–III breast cancer prescribed ET were identified from the National Cancer Registry Ireland (*N* = 2423) and invited to complete a questionnaire. A theoretically based model of non-adherence was developed using PAPA to examine inter-relationships between the 14 TDF domains of behaviour change and self-reported non-adherence. Structural equation modelling (SEM) was used to test the model.

**Results:**

A total of 1606 women participated (response rate = 66%) of whom 395 (25%) were non-adherent. The final SEM with three mediating latent variables (LVs) (PAPA Perceptions: TDF domains, *Beliefs about Capabilities,* *Beliefs about Consequences*; PAPA Practicalities: TDF domain, *Memory, Attention,* *Decision Processes* *and Environment*) and four independent LVs (PAPA Perceptions: Illness intrusiveness; PAPA Practicalities: TDF domains, *Knowledge,* *Behaviour Regulation*; PAPA External Factors: TDF domain, *Social Identity*) explained 59% of the variance in non-adherence and had an acceptable fit (*χ*^2^(334) = 1002, *p* < 0.001; RMSEA = 0.03; CFI = 0.96 and SRMR = 0.07) *Knowledge* had a significant mediating effect on non-adherence through *Beliefs about Consequences* and *Beliefs about Capabilities*. Illness intrusiveness had a significant mediating effect on non-adherence through *Beliefs about Consequences*. *Beliefs* *about Consequences* had a significant mediating effect on non-adherence through *Memory, Attention, Decision Processesg and Environment*.

**Conclusions:**

By underpinning future interventions, this model has the potential to improve ET adherence and, hence, reduce recurrence and improve survival in breast cancer.

**Supplementary Information:**

The online version contains supplementary material available at 10.1007/s00520-023-07658-x.

## Introduction

With contemporary multidisciplinary management, survival in women with breast cancer has increased steadily and 5-year net survival in high-income countries is now approaching 85–90% [1]. Approximately 75% of breast cancers are oestrogen receptor–positive (ER +) and 5–10 years of adjuvant endocrine therapy (ET) halves the risk of recurrence and reduces mortality by one-third in ER + women [2, 3]. However, despite proven clinical efficacy many women do not take ET as prescribed [4]. In routine clinical settings, 28–59% of women do not take their prescribed dosage and 38–60% discontinue treatment by 5 years [4]. Discontinuing or taking less than 80% of prescribed doses (henceforth, non-adherence) has been associated with a 3-fold increased risk of cancer recurrence and raised risk of mortality [5–7].

Recently, studies have explored modifiable determinants of ET non-adherence, with the aim of better supporting adherence [8]. An integrative review found that ET non-adherence is associated with several psychosocial factors, including side effects, lack of medication-taking routine, lack of social support, negative ET beliefs, low self-efficacy and dissatisfaction with patient-provider relationship [8]. However, no studies to date have examined inter-relationships between these determinants and how they might predict (non-) adherence. A further limitation of previous research has been the lack of incorporation of theoretical frameworks to help understand how these modifiable determinants predict non-adherence [9]. A recent meta-analysis examining interventions to improve ET adherence identified 16 studies (4 randomised controlled trials) and found that most interventions were educational only and did not improve adherence [10, 11]. Interventions also lacked a theoretical basis, and none had systematically identified and addressed modifiable determinants of adherence in their population of interest, despite these steps being key in complex intervention development [10].

While several specific health psychology theories have relevance to medication adherence, broader theoretical frameworks that may have relevance to adherence support have recently been adopted in the literature and clinical practice [12]. The Theoretical Domains Framework (TDF) and Perceptions and Practicalities Approach (PAPA) can both be used to identify root causes of non-adherence [13, 14]. The TDF distils numerous psychological theories into 14 domains for understanding and changing behaviour, with each domain defined as a group of related theoretical constructs (e.g. the domain *Social Influences* includes social support, group norms and others) [13]. The NICE Medicines Adherence Guidelines recommend PAPA as an overarching framework for developing and providing adherence support [14]. PAPA specifies the “minimum ingredients” for developing adherence interventions, namely specific Perceptions (e.g. beliefs about the treatment and condition) and Practicalities (e.g. capabilities and resources) which influence an individual’s motivation and ability to take their medication [15]. Both the TDF and PAPA can be linked to evidence-based behaviour change techniques (BCTs) which enable adherence interventions to be designed to include techniques which target specific determinants of non-adherence [16].

Our study aims to address the current gaps in evidence by: (i) using the TDF to measure key modifiable determinants of ET non-adherence and (ii) examining the inter-relationships between these modifiable determinants and non-adherence, based on PAPA, using data from a national survey of women with stages I–III breast cancer prescribed ET in Ireland.

## Methods

### Study population

**Fig. 1:**
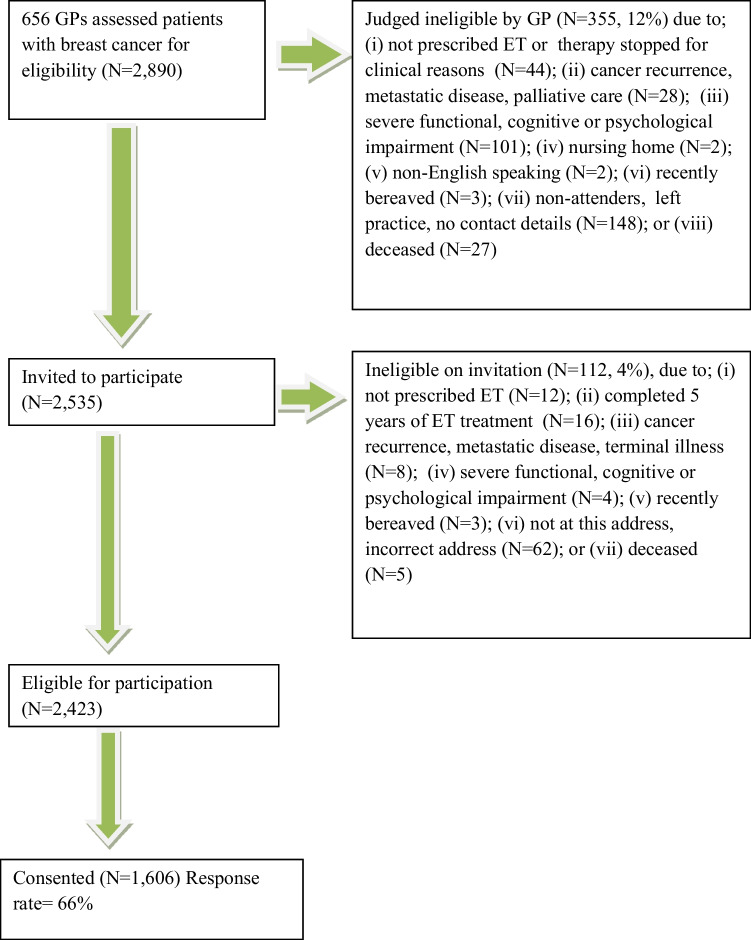
Number of participants at each stage of the study

This study was a national population-based survey of women with stages I–III breast cancer prescribed ET in Ireland. Eligible women were identified from the records of the population-based National Cancer Registry Ireland and were: (i) aged ≥ 18 years; (ii) had a diagnosis of stages I–III, estrogen (ER) or progesterone (PR) receptor positive breast cancer during 01/07/2009-–30/06/2014; (iii) received tumour-directed surgery; (iv) were prescribed adjuvant ET (selective estrogen receptor modulator, SERM; aromatase inhibitor, AI) within 1 year of breast cancer diagnosis and up to 5 years and were (v) alive. Women were excluded if they had previously been diagnosed with another invasive cancer other than non-melanoma skin cancer (*N* = 2890 eligible women). GPs (*N* = 656) screened women for any medical reasons that they should not be invited to take part in the study (e.g. cancer recurrence, metastatic disease, palliative care, deceased) and 355 (12%) were considered to be ineligible. In total, 2535 eligible women were invited, by post, to self-complete a questionnaire measuring; (i) socio-demographics; (ii) modifiable determinants of ET non-adherence and; (iii) non-adherence (Fig. 1). Ethical approval was granted by the Irish College of General Practitioners. Informed consent was obtained from all individual participants included in the study.

### Outcome measure (non-adherence)

ET non-adherence was measured using the Adherence Taxonomy and included *implementation*, the extent to which a patient’s actual dosing regimen corresponded to their prescribed dosing regimen, and *discontinuation*, defined as when the patient stops taking their medication, and no more doses are taken thereafter [17]. Participants were asked whether they were; (i) currently taking ET as prescribed (*adherent*) or; (ii) taking ET but regularly missing doses (*suboptimal implementation*) or; (iii) had stopped taking ET (*discontinuation*). Participants who had discontinued ET were also asked when they stopped and who made the decision to stop (i.e. themselves alone, their oncologist/healthcare provider (HCP) or a shared patient-provider decision). For analysis, adherence was measured as a binary outcome (adherent/non-adherent) with non-adherence including both *suboptimal implementation* and *discontinuation* (without oncologist/HCP agreement).

### Theoretical frameworks and the modifiable determinants of ET non-adherence

Modifiable determinants of ET non-adherence were measured using a TDF-based questionnaire which included questions which tapped into eight TDF (henceforth, TDF-8) domains shown in past work to influence ET adherence (*Knowledge, Social Identity, Social Influences, Beliefs about Capabilities, Beliefs about Consequences, Intentions Goals and Reinforcement, Behaviour Regulation* and *Memory, Attention, Decision Processes and Environment*) and their associated theoretical constructs [18]. Validated scales were included for each construct within each domain (e.g. the construct beliefs and concerns about medications within the domain *Beliefs about Consequences* were assessed by the Beliefs about Medicines Questionnaire (BMQ)) [18, 19]. A measure of coping with ET side effects was added to the domain *Beliefs about Capabilities* for this study (based on Brief COPE [20])*.*

**Fig. 2:**
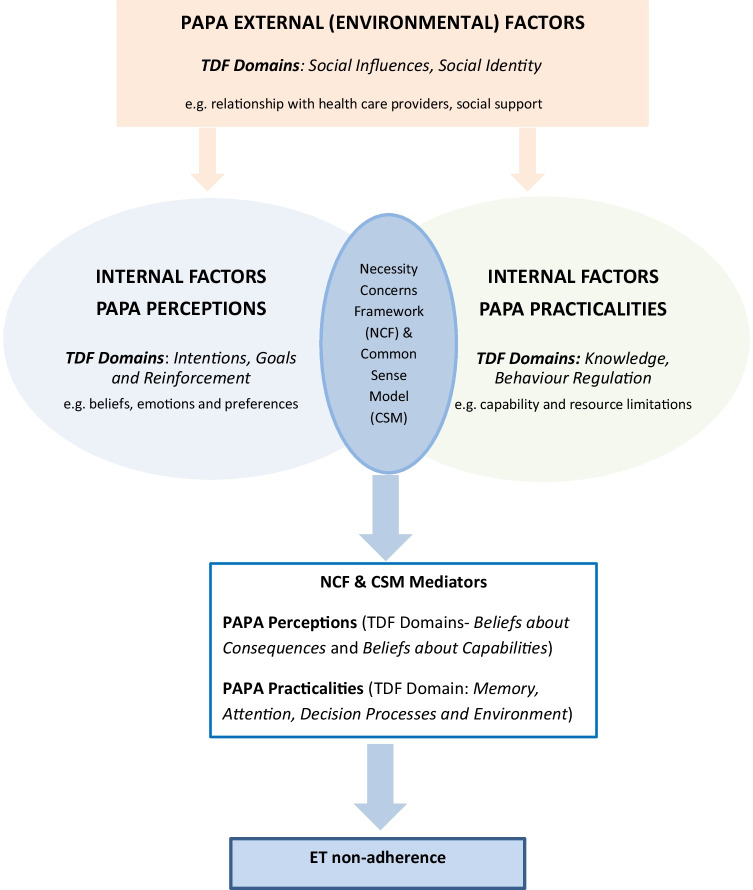
The hypothesised relationship between the TDF and PAPA and the modifiable determinants of ET non-adherence

PAPA was used to examine inter-relationships between the modifiable determinants of ET (TDF domains) and non-adherence. At the core of PAPA is a symbiotic relationship between the Necessity Concerns Framework (NCF) and Leventhal’s Common-Sense Model of self-regulation (CSM) [15]. The CSM proposes that patients hold implicit common-sense beliefs about their illness for making sense of, and coping with, their illness. The NCF proposes that patients undertake a cost–benefit analysis of their medication, weighing up the necessity of their prescribed medication against concerns regarding potential adverse effects [15]. We hypothesised that the TDF domains (*Beliefs about Capabilities, Beliefs about Consequences, Intentions, Goals and Reinforcement)* measuring PAPA Perceptions and the TDF domains (*Behaviour Regulation, Knowledge, Memory, Attention, Decision processes and Environment*) measuring PAPA Practicalities may mediate each other and influence non-adherence, according to the NCF and CSM [21]. As well as PAPA Perceptions and Practicalities (internal factors), PAPA also recognises external (environmental) factors such as quality of communication with HCPs (TDF domain, *Social Identity*) and social support (TDF domain, *Social Influences*) [15] (Fig. 2).

### Data analysis

Descriptive statistics including medians (inter-quartile range, IQR) and proportions were calculated for all the potentially modifiable determinants of ET non-adherence measured by TDF-8 and PAPA. Relationships between these individual determinants and non-adherence were examined using chi-square tests for categorical variables and non-parametric Wilcoxon rank sum tests for continuous variables, with Bonferroni corrections (*p* < 0.001), using Stata Version 16 (Stata Corporation, College Station, TX, USA).

#### Confirmatory Factor Analysis (CFA) model: measuring determinants of ET non-adherence using the TDF

CFA was used to examine whether the items measuring the determinants of non-adherence were a good fit to the TDF-8 model using weighted least square estimates (WLSMV) [22]. WLSMV is a robust estimator which does not assume normally distributed variables and provides the best option for modelling categorical or ordered data on a probit scale [22]. Recommendations for assessing adequate model fit were applied, namely root mean square error of approximation (RMSEA) < 0.06 and comparative fit index (CFI) > 0.90 and standardised root mean squared residual (SRMR) < 0.08. The model fit was improved by inspecting modification indices and standardised residuals and by assessing each domain/factor and its related items. Items/measures that did not sufficiently load onto domains and had coefficients (*β*) < 0.30 and/or large standardised residuals were removed. Inter-item correlations were used to test for internal consistency between items included in the factor analysis, with values above 0.15 to 0.50 considered the optimal range [23]. Discriminant validity was assessed using Fornell and Larkner’s tests [24].

#### Structural Equation Model (SEM): examining inter-relationships between modifiable determinants and non-adherence using PAPA

The CFA model was extended to form SEM by specifying causal paths between the TDF domains and ET non-adherence based on PAPA [21]. Indirect pathways were added to the SEM model to establish if; (i) the effect of the TDF domains within PAPA Perceptions and PAPA Practicalities on non-adherence was mediated by beliefs about the treatment and condition and; (ii) the influence of these beliefs on non-adherence were in turn mediated by practicalities (e.g. capacity, resource limitations). The SEM contained one dependent variable (non-adherence), three mediating latent variables (LVs) (PAPA Perceptions: TDF domains, *Beliefs about Capabilities* and *Beliefs about Consequences*; PAPA Practicalities: TDF domain, *Memory, Attention, Decision Processes and Environment*) and five independent LVs (PAPA Perceptions: *Intentions, Goals and Reinforcement*, PAPA Practicalities: *Knowledge, Behaviour Regulation* and PAPA External Factors: *Social Identity, Social Influences*). The SEM model fit was assessed and the significance of indirect effects was determined by calculating probit path coefficients and bootstrapped confidence intervals for each parameter. The CFA and SEM analysis was undertaken using Mplus Version 8.5.

## Results

### Descriptive statistics

In total, 1606 women with stages I–III breast cancer prescribed ET completed the questionnaire (response rate = 66%: Fig. 1). The mean age was 60 years (SD = 10.2, range 34–86 years). Three-quarters of women were married or in a long-term relationship (73.4%) and almost half (45.3%) had completed third level (degree) or post-graduate education. In total, 1211 (75%) women were classified as adherent on self-report, 180 (11%) women were classified as having suboptimal implementation and 215 (14%) women were classified as having discontinued treatment (total non-adherent; *N* = 395, 25%).

Table S1 describes the characteristics of the modifiable determinants of ET non-adherence in the study population according to the TDF-8 and PAPA. Women who were classified as non-adherent reported significantly lower self-efficacy than adherent women. Significantly lower proportions of non-adherent women used coping skills such as positive reinterpretation and growth, active coping and acceptance. Non-adherent women had a significantly lower perceived utility in taking ET, a smaller necessity-concerns (NCF) differential in their ET beliefs and more negative outcome expectancies. They also reported lower motivation to take ET and a higher interference between taking ET and aspects of their day-to-day life. In terms of taking ET, non-adherent women reported significantly less use of action and coping planning and self-monitoring, as well as difficulties remembering to take their ET, especially when there were changes/interruptions to their normal routine.

#### CFA model: measuring determinants of ET non-adherence using the TDF

The initial CFA based on the TDF-8 model did not fit adequately (*χ*^2^(853) = 4344, *p* < 0.001; RMSEA = 0.05; CFI = 0.83; SRMR = 0.06). Per the standard approach, the participant sample (*N* = 1606) was randomly split in two, and a CFA was undertaken again on half the sample, having examined the individual items and measures within each of the eight domains. The domain *Social Influences* was removed, as the constructs did not sufficiently load onto the domain (Table S1). The domain *Beliefs about Capabilities* lacked discriminant validity so the measures of self-efficacy and resilience were removed and three measures of coping skills were retained (positive reinterpretation and growth, active coping, acceptance). Similarly, the domain *Beliefs about Consequences* was refined to include beliefs about medication, outcome expectancies and goal conflict and facilitation*.* Measures of intentions and motivation were removed from the domain *Intentions, Goals and Reinforcement* as these measures also loaded onto several domains, and the domain was refined to measure the construct of Illness Intrusiveness only and renamed accordingly. The measure of general knowledge was removed from the domain *Knowledge*; measures of action control were removed from the domain *Behaviour Regulation*; the measure of habit strength was removed from the domain *Memory, Attention, Decision Processes and Environment.*

**Fig. 3:**
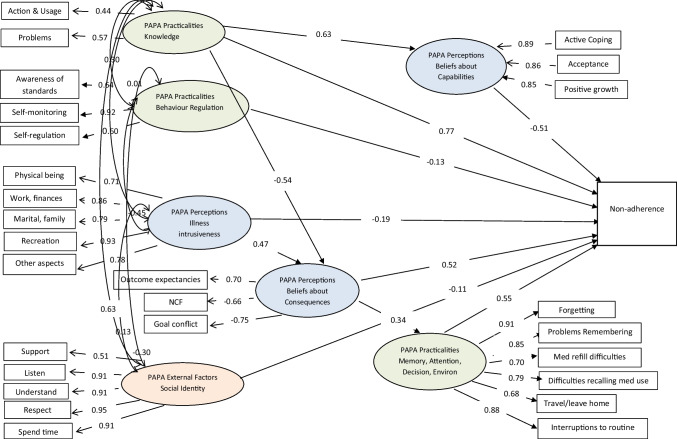
SEM probit analysis of PAPA and TDF and ET non-adherence

Our final model consisted of six TDF (henceforth, TDF-6) domains plus Illness Intrusiveness and was a reasonable fit (random sample: *N* = 803; *χ*^2^(303) = 580, *p* < 0.001; RMSEA = 0.03; CFI = 0.96; SRMR = 0.06; all participants: *N* = 1606; (*χ*^2^(303) = 681, *p* < 0.001; RMSEA = 0.03; CFI = 0.97; SRMR = 0.05) (Fig. 3). All TDF domains demonstrated adequate levels of internal consistency (*r* > 0.15 and *r* < 0.50) and displayed discriminant validity (Table S2).

#### SEM: examining inter-relationships between the modifiable determinants and non-adherence using PAPA

The CFA measurement model was extended to form a SEM including the dependent variable (non-adherence), three mediating LVs (PAPA Perceptions: *Beliefs about Capabilities, Beliefs about Consequences* and PAPA practicalities: *Memory, Attention, Decision Processes and Environment*) and four independent LVs (PAPA Perceptions: Illness Intrusiveness, PAPA Practicalities: *Knowledge, Behaviour Regulation* and PAPA External Factors:* Social Identity*). The SEM demonstrated an acceptable fit to the data (*χ*^2^(334) = 1002, *p* < 0.001; RMSEA = 0.03; CFI = 0.96; SRMR = 0.07) and explained 59% of the variance in non-adherence (*R*^2^ = 0.59). (Table 1; Fig. 3).

**Table 1:** Probit path coefficients and 95% confidence intervals (CI) of direct effects on mediating domains and non-adherence

TDF domains	Mediating domains			Dependent variable (non-adherence)
	*Beliefs about Consequences*	*Beliefs about Capabilities*	*Memory, Attention, Decision Processes and Environment*	Non-adherence
Internal Factors Perceptions	Probit path coefficients (95% CI)	Probit path coefficients (95% CI)	Probit path coefficients (95% CI)	Probit path coefficients (95% CI)
*Beliefs about Capabilities* *Beliefs about Consequences* Illness Intrusiveness	- - 0.47 (0.38, 0.56)*	- - -	- 0.34 (0.26, 0.41)* -	− 0.51(− 0.80, − 0.27)* 0.52 (0.22, 0.98)* − 0.19 (− 0.39, − 0.04)*
Internal Factors PAPA — Practicalities				
*Knowledge* *Behaviour Regulation* *Memory, Attention, Decision Processes and Environment*	− 0.54 (− 0.65, − 0.44)* - -	0.63 (0.51, 0.74)* - -	- - -	0.77 (0.32, 1.36)* − 0.13 (− 0.25, − 0.04)* 0.55 (0.47, 0.63)*
External (Environmental) Factors PAPA				
*Social Identity*	-	-	-	− 0.11 (− 0.28, 0.05)

^*^p < 0.05

There was a significant direct positive relationship between *Beliefs about Consequences* (*β* = 0.52, 95% CI 0.22, 0.98), *Memory, Attention, Decision Processes and Environment* (*β* = 0.55, 95% CI 0.47, 0.63) and *Knowledge* (*β* = 0.77, 95% CI 0.32, 1.36) and non-adherence. *Beliefs about* *Capabilities* (*β* =  − 0.51, 95% CI − 0.80, − 0.27), *Behaviour Regulation* (*β* =  − 0.13, 95% CI − 0.25, − 0.04) and the construct Illness Intrusiveness (*β* =  − 0.19, 95% CI − 0.39, − 0.04) had a significant direct negative relationship with non-adherence. There was no significant direct relationship between *Social* *Identity* and non-adherence. *Knowledge* had a significant direct negative effect on *Beliefs about Consequences* (*β* =  − 0.54, 95% CI − 0.65, − 0.44) and a positive effect on *Beliefs about Capabilities* (*β* = 0.63, 95% CI 0.51, 0.74). Illness Intrusiveness had a significant direct positive effect on *Beliefs about Consequences* (*β* = 0.47, 95% CI 0.38, 0.56) and *Beliefs about Consequences* had a significant direct positive effect on *Memory, Attention, Decision Processes and Environment* (*β* = 0.34, 95% CI 0.26, 0.41).

For the indirect effects, *Knowledge* (*β* =  − 0.28, 95% CI − 0.60, − 0.11) and Illness Intrusiveness (*β* = 0.25, 95% CI 0.11, 0.46) had a significant mediating effect on non-adherence through *Beliefs* *about Consequences*, explaining 74% of the variance in *Beliefs about Consequences* (*R*^2^ = 0.74). *Knowledge* (*β* =  − 0.32, 95% CI − 0.57, − 0.15) had a significant mediating effect on non-adherence through *Beliefs about Capabilities*, explaining 40% of the variance in *Beliefs about Capabilities* (*R*^2^ = 0.40). *Beliefs about Consequences* (*β* = 0.19, 95% CI 0.14, 0.24) had a significant mediating effect on non-adherence through *Memory, Attention, Decision Processes and Environment*, explaining 11% of the variance in *Memory, Attention, Decision Processes and Environment* (*R*^2^ = 0.11).

## Discussion

### Summary of main findings

This study used the TDF to measure key modifiable determinants of ET non-adherence and examined inter-relationships between these modifiable determinants and non-adherence using PAPA. Significant direct and indirect associations were found between the TDF domains in PAPA Perceptions and PAPA Practicalities and non-adherence; explaining 59% of the variation in ET non-adherence. While previous studies have identified modifiable determinants associated with non-adherence, they have not examined inter-relationships between them and how they influence non-adherence.

The domain *Beliefs about Consequences* (PAPA Perceptions), consisting of higher negative outcome expectancies (e.g. diminished quality of life) and a lower NCF differential (perceived low ET necessity and high concerns), was directly associated with non-adherence. This is consistent with previous research, which has indicated that negative beliefs about ET necessity [25] and perceiving severe consequences from taking ET are associated with lower adherence [9]. The domain *Belief about Capabilities* (PAPA Perceptions) was also directly associated with adherence. Women who used coping skills such as positive reinterpretation and growth, active coping and acceptance more often adhered. A mixed-methods study found acceptance to be the most frequently used coping strategy for women experiencing stressful events or problems while taking ET and that it was associated with treatment endurance [26].

Illness intrusiveness (PAPA Perceptions) was directly associated with *Beliefs about Consequences* and directly and indirectly associated with non-adherence through mediating *Beliefs about Consequences*. Perceiving that ET may disrupt your lifestyle, activities and interests was associated with a lower NCF differential and higher negative outcome expectancies, leading to non-adherence (mediating effect). For many breast cancer survivors, ET adherence is perceived as a balancing act between quality and quantity of life [27]. Side effects such as arthralgia, hot flushes, weight gain and loss of libido can substantially reduce quality of life [28] but women may persevere with treatment due to strong beliefs in the efficacy of ET and fear of recurrence [27, 29]. Research on chronic disease in general has shown that people with more negative attitudes towards medication at baseline are more likely to misattribute non-medication symptoms as adverse effects [30]. Our findings suggest that discussions around the prevalence of side effects and their potential impact on lifestyle at treatment initiation may help inform more realistic expectations around the adverse impact of ET on lifestyle and support adherence.

The domain *Knowledge* (PAPA Practicalities) was directly associated with *Beliefs about Consequences* (PAPA Perceptions) and *Beliefs about Capabilities* (PAPA Perceptions) and indirectly associated with non-adherence through mediating *Beliefs about Consequences* (PAPA Perceptions) and *Beliefs about Capabilities* (PAPA Perceptions)*.* Within our study population, women who reported they were informed about why they were taking ET, how it works, their risk of side effects and what to do if they experienced side effects had a higher NCF differential (perceived high ET necessity and low concerns) and fewer negative outcome expectancies, better use of coping skills and greater adherence (mediating effect). The information women are provided about their diagnosis, hormone receptor status and ET is often insufficient and lack of knowledge has been shown to predict non-adherence [31]. One study reported that, in two-thirds of consultations, the ET treatment decision was taken by HCPs before the woman had received any information or had the opportunity to consider treatment benefits or concerns [32]. Some HCPs have difficulty discussing ET side effects with patients, measures to ameliorate symptoms or the option to switch to another treatment [33]. Equally, some non-adherent women report feeling unprepared for side effects and having limited information on management strategies from HCPs [34]. In a US study, more than one-third of patients did not discuss side effects with their HCPs despite reporting good doctor-patient rapport; elsewhere, women who had difficulties asking HCPs for more information were more likely to report side effects [35].

Our findings indicate firstly, that improving the content and timing of information may reduce women’s decisional conflict and increase certainty regarding their choices around taking ET and managing side effects and secondly, that ET side effects should be routinely assessed and discussed during HCP consultations. Interventions are available to mitigate the symptom burden, increase quality of life and enable more accurate symptom attribution for those with negative perceptions of medication [36]. Moreover, the findings confirm that while knowledge is an important determinant of ET adherence, it is related to other determinants. Thus, knowledge and information by themselves are unlikely to be effective in improving adherence, which may explain the largely null findings of past interventions in this area [10].

Within the domain *Memory, Attention, Decision Processes and Environment* (PAPA Practicalities) difficulty remembering to take ET, especially during changes/interruptions to normal routines, was associated with non-adherence. Active self-monitoring and self-regulation of medication taking within the domain *Behaviour Regulation* (PAPA Practicalities) were also associated with adherence. These findings confirm that the development of robust medication–taking routines could help women take ET on a daily basis. In terms of specific strategies, previous studies reported that reminders and visual cues were helpful [29].

*Beliefs about Consequences* (PAPA Perceptions) was also directly associated with *Memory, Attention, Decision Processes and Environment* (PAPA Practicalities) and indirectly associated with non-adherence through mediating domain *Memory, Attention, Decision Processes and Environment* (PAPA Practicalities). These findings suggest that women who do not perceive their ET to be a necessity/important are more likely to forget to take it. While some women may benefit from strategies that promote habitual medication taking, this suggests that others may need more active behavioural approaches to address beliefs, such as cognitive behavioural therapy or motivational interviewing, in order to prioritise ET and hence adherence [37]. Most clinical interventions for medication adherence to date have not included behavioural approaches [38].

### Limitations

Non-adherence was measured using self-report which may over-estimate adherence rates [39]. However, self-report adherence measures have at least modest to high concordance with electronic monitoring of adherence [40] and have been shown to be associated with disease outcomes [41]. The cross-sectional design limits the interpretation of the results, and longitudinal studies are needed to confirm these relationships. Demographic and clinical determinants were not investigated in the current model. Determinants such as age, ethnicity, out of pocket costs and the use of antidepressants have previously been shown to be associated with ET non-adherence [42]. Health beliefs have been shown to differ according to sociodemographic variables in breast cancer, with minority women perceiving less risk of recurrence and greater barriers to treatment [43]. Future research should explore if the identified determinants of non-adherence in the existing model differ across sociodemographic and clinical covariates, e.g. age, ethnicity, breast cancer prognosis and treatments [44].

### Implications and conclusions

Our findings suggest that ET adherence can be perceived as interrelated appraisals and behaviours around the ET necessity for prevention of recurrence versus concerns about adverse effects, with gaps in knowledge, lack of coping skills to allay fears and deal with potential side effects and poor medication management techniques/low prioritisation all contributing to reduced adherence. This, in turn, implies that adherence support for ET will be more effective if a multi-faceted approach is applied that addresses both PAPA Perceptions (e.g. beliefs and outcomes expectancies) and PAPA Practicalities (e.g. capability and resources) to non-adherence concurrently.

Breast cancer care is also changing, with treatment becoming more personalised involving less use of chemotherapy and radiotherapy and women expected to self-manage [45, 46]. Self-management interventions based on this multi-faceted theoretical approach are needed. Some progress has been made recently with self-management-based multi-faceted interventions using digital technologies showing promising results in pilot studies [47, 48]. Using the model developed here to underpin the development of future interventions of this type is likely to have the greatest potential to improve ET adherence and, hence, reduce recurrence and improve survival in breast cancer.

## Supplementary Information

Below is the link to the electronic supplementary material.

Supplementary file1 (DOCX 35.5 KB)

## Data Availability

Due to the nature of this research, participants of this study did not agree for their data to be shared publicly, so supporting data is not available for general use.
