# Supplementary material for: Informing interventions to improve uptake of adjuvant endocrine therapy in women with breast cancer: a theoretical-based examination of modifiable influences on non-adherence
Source: Support Care Cancer. 2023 Mar 4;31(3):200. doi: 10.1007/s00520-023-07658-x (PMC9985562; doi:10.1007/s00520-023-07658-x)
Supplement: Supplementary file 1 — Supplementary file1 (DOCX 35.5 KB) [file 520_2023_7658_MOESM1_ESM.docx]

**Table S1:** Characteristics of the modifiable determinants of ET non-adherence grouped by TDF-8 domains and PAPA: medians, inter-quartile ranges (IQR), numbers (N) and percentages (%)

| **Modifiable determinants** | **Adherent women** | **Non-adherent women** | **p value** |
| --- | --- | --- | --- |
| **Internal factors; PAPA - Perceptions** |  |  |  |
| ***Beliefs about Capability*** | *Median (IQR)* | *Median (IQR)* |  |
| Action self-efficacy* | 10 (9, 10) | 10 (8, 10) | p<0.001 |
| Maintenance self-efficacy* | 10 (9, 10) | 10 (8, 10) | p<0.001 |
| Recovery self-efficacy* | 10 (9, 10) | 10 (7, 10) | p<0.001 |
| Resilience | 3.5 (3, 4) | 3.7 (3, 4) | p=0.05 |
| *Coping skills^**^* | *N (%)* | *N (%)* |  |
| Positive reinterpretation and growth* | 896 (92.7) | 255 (81.7) | p<0.001 |
| Active coping* | 968 (97.8) | 296 (97.8) | p<0.001 |
| Planning | 734 (76.6) | 227 (73.5) | p=0.26 |
| Seeking emotional support | 715 (73.4) | 232 (73.2) | p=0.94 |
| Seeking instrumental support | 706 (73.5) | 227 (71.8) | p=0.55 |
| Spirituality* | 669 (69.2) | 183 (57.0) | p<0.001 |
| Acceptance* | 934 (95.6) | 289 (89.2) | p<0.001 |
| Venting of emotions | 381 (39.4) | 117 (37.1) | p=0.48 |
| Humour | 401 (41.4) | 142 (44.7) | p=0.31 |
| Reflection | 818 (83.6) | 252 (78.5) | p=0.04 |
| ***Beliefs about Consequences*** | *Median (IQR)* | *Median (IQR)* |  |
| Perceived severity of breast cancer | 9 (7, 10) | 8 (6, 10) | p=0.05 |
| Perceived susceptibility to breast cancer | 10 (9, 12) | 11 (8, 12) | p=0.55 |
| Perceived utility of adhering* ^***^ | 15 (11, 17) | 16 (11, 19) | p<0.001 |
| Beliefs about medication–necessity and concerns (NCF) differential* ^****^ | 3 (0, 6) | 1 (-2, 5) | p<0.001 |
| Outcome expectancies- impact on daily life if take ET* | 11 (9, 12) | 12 (9, 13) | p<0.001 |
| Outcome expectancies – likelihood of recurrence - if take ET | 2 (1,3) | 3 (2, 3) | p=0.001 |
| Outcome expectancies – likelihood of recurrence - if don’t take ET | 5 (4, 6) | 5 (4, 6) | p=0.69 |
| Illness perceptions- recurrence affect your life | 10 (8, 10) | 9 (7, 10) | p=0.10 |
| Concern about recurrence | 1.8 (1.3, 2) | 1.8 (1.3, 2.3) | p=0.74 |
| ***Intentions, goals and reinforcement*** | *Median (IQR)* | *Median (IQR)* |  |
| Intention to take ET* | 19 (16, 20) | 18 (16, 20) | p<0.001 |
| Autonomous motivation* | 6.2 (5.3, 7) | 5.8 (4.7, 6.5) | p<0.001 |
| Introjected regulation* | 5.5 (3, 7) | 4.5 (2.5, 6) | p<0.001 |
| External regulation | 2.3 (1.3, 3) | 2.3 (1.3, 3) | p=0.73 |
| Amotivation | 3 (2, 4.3) | 3 (2, 4) | p=0.003 |
| Adapted Illness Instrusiveness Ratings- Physical well-being* | 1 (0, 3) | 2 (0, 3.5) | p<0.001 |
| Adapted Illness Instrusiveness Ratings- Work and finances | 0.5 (0, 2.5) | 1 (0, 3) | p=0.002 |
| Adapted Illness Intrusiveness Ratings- Marital, sexual and family relationships* | 0.7 (0, 2.7) | 1.3 (0, 3.5) | p<0.001 |
| Adapted Illness Intrusiveness Ratings- Recreation and social relationships* | 0.3 (0, 2) | 1 (0, 3) | p<0.001 |
| Adapted Illness Intrusiveness Ratings- Other aspects of life | 0 (0, 1) | 0 (0, 1.8) | p=0.001 |
| Goal conflict and facilitation* | 2.3 (2, 2.8) | 2.5 (2, 3.3) | p<0.001 |
| **Internal factors; PAPA Practicalities** |  |  |  |
| ***Knowledge*** | *Median (IQR)* ^a^ | *Median (IQR)* ^a^ |  |
| Action and usage | 7 (5, 8) | 7 (4,8) | p=0.41 |
| Potential problems* | 3 (1, 6) | 2 (0, 6) | p<0.001 |
| General knowledge | 3 (2, 3.5) | 3 (2, 3) | p=0.59 |
| ***Behaviour Regulation*** | *Median (IQR)* | *Median (IQR)* |  |
| Action planning* | 3.3 (3, 4) | 3 (3, 4) | p<0.001 |
| Coping planning* | 3 (2.5, 3.3) | 3 (2.3, 3) | p<0.001 |
| Action control-awareness of standards | 2.5 (2.5, 3) | 2.5 (2, 3) | p=0.39 |
| Action control-self-monitoring* | 3 (2.5, 3.5) | 3 (2.5, 3) | p<0.001 |
| Action control-self-regulatory effort | 3 (3, 4) | 3 (2.5, 3) | p=0.90 |
| ***Memory, attention, decision making and environment*** | *N (%)* | *N (%)* |  |
| Have you ever forgotten to take your ET (Yes) * | 291 (24.6) | 198 (68.5) | p<0.001 |
| Problems remembering to take ET (Yes) * | 122 (10.4) | 109 (38.4) | p<0.001 |
| Forgetting/difficulties remembering to get prescription refilled (Yes) * | 38 (3.2) | 36 (12.5) | p<0.001 |
| Forgetting/difficulties recalling medication usage (Yes) * | 107 (9.1) | 69 (23.9) | p<0.001 |
| Environmental Context- forget when travelling or leaving home (Yes) * | 86 (7.3) | 59 (20.4) | p<0.001 |
| Environmental Context- forget when interruptions to normal routine (Yes) * | 171 (14.5) | 127 (43.9) | p<0.001 |
| Habit strength-history of repetition, automaticity (lack of control and awareness, efficiency) * (Median, IQR) | 3.3 (2.8, 4) | 3 (2.3, 3.5) | p<0.001 |
| **External (environmental) factors**  **PAPA** |  |  |  |
| ***Social Influences*** | *Median (IQR)* | *Median (IQR)* |  |
| Support and barriers to adherence | 10 (9, 12) | 10 (9, 12) | p=0.004 |
| Injunctive norms | 7 (6.3, 7) | 7 (6, 7) | p=0.01 |
| Descriptive norms | 7 (5.5, 7) | 6 (5, 7) | p=0.001 |
| ***Social Identity*** | *N (%)* | *N (%)* |  |
| Support oncologist (right level) | 1,002 (83.5) | 309 (79.4) | p=0.07 |
| Support from oncology services- one main point of contact/support | 870 (72.9) | 262 (66.8) | p=0.02 |
| Relationship with oncologist- listening (Yes) | 1,019 (88.3) | 318 (83.3) | p=0.01 |
| Relationship with oncologist- understanding (Yes) | 1,000 (86.8) | 311 (81.8) | p=0.02 |
| Relationship with oncologist- respect (Yes) | 1,028 (89.2) | 325 (85.3) | p=0.04 |
| Relationship with oncologist- time (Yes) | 959 (83.3) | 301 (78.6) | p=0.04 |

Details of the measures and scales used for each modifiable determinant can be found in the questionnaire development paper (18)

*The relationships between these individual determinants and non-adherence were examined using chi-square tests for categorical variables and non-parametric Wilcoxon Rank Sum tests for continuous variables, with Bonferroni corrections (p<0.001)

^**^ A new measure of coping skills based on the Brief COPE scale was added to the domain *Beliefs about Capabilities* (20)

^***^Higher score- perceived lower utility of adhering to ET

^****^A positive differential means the patient perceives that the benefits (necessity) of their medication outweigh the costs (concerns).

**Table S2:** Discriminant validity and internal consistency of each TDF domain

| **TDF Domain** | **AVE (average variance extracted)*** | **Average inter-item correlation**** |
| --- | --- | --- |
| Knowledge | 0.64 | 0.31 |
| Social Identity | 0.73 | 0.44 |
| Beliefs about Capability | 0.75 | 0.38 |
| Beliefs about Consequences | 0.52 | 0.50 |
| Behaviour Regulation | 0.53 | 0.49 |
| Memory, attention, decision making and environment | 0.65 | 0.34 |

Details of the measures and scales used for each TDF domain can be found in the questionnaire development paper (18)

* AVE can be used to gauge discriminant validity. If the squared (error-disattenuated or structural equation model) correlation between two LV's (domains) is less than either of their individual AVE's this suggests the LV's (domains) each have more internal (extracted) variance than variance shared between the LV's (domains). If this is true for the target LV (domain) and all the other LV's (domains), this suggests the discriminant validity of the target LV (domain)(24)

**Inter-item correlations were used to test for internal consistency, with values above 0.15 to 0.50 being the optimal range
